# Effects of Using a Perforated Spoon on Salt Reduction When Consuming Ramen Noodles: A Randomized Crossover Study of Japanese Male University Students

**DOI:** 10.3390/nu15132864

**Published:** 2023-06-24

**Authors:** Maiko Sugimoto, Eri Tajiri, Nana Nakashima, Tatsuaki Sakamoto

**Affiliations:** 1 Graduate School of Environmental & Symbiotic Sciences, Prefectural University of Kumamoto, 3-1-100, Tsukide, Higashi-ku, Kumamoto 862-8502, Japan; g2270008@pu-kumamoto.ac.jp (M.S.); e-tajiri@pu-kumamoto.ac.jp (E.T.);; 2 Public Health and Disease Prevention Division, Ariake Public Health Center, Kumamoto Prefectural Government, 1004-1, Iwasaki, Tamana, Kumamoto 865-0016, Japan

**Keywords:** salt, salt reduction, diet, nudge, ramen, Japan

## Abstract

Salt reduction is a public health priority for the Japanese population. We focused on the effect of salt reduction by changing eating utensils to reduce salt consumption. As a test meal, we used ramen, which is commonly eaten by Japanese individuals and has a high salt content. In this randomized crossover study, we hypothesized that eating ramen with a perforated spoon would reduce the quantity of ramen soup and salt consumed compared to using a regular spoon without holes. Soup intake, after-meal fullness, and deliciousness were compared between eating with chopsticks and a regular spoon, and with chopsticks and a perforated spoon. In total, 36 male university students (mean age, 20.7 [standard deviation, 1.8] years) were included in the analyses. The median salt intake (25th and 75th percentiles) was significantly lower with perforated spoons (1.8 [1.5, 4.3] g) than with regular spoons (2.4 [1.8, 4.8] g; *p* = 0.019). There were no significant differences in after-meal fullness or deliciousness for both spoon conditions (*p* > 0.05). For young men, the soup intake when eating ramen with a perforated spoon was lower than that with a regular spoon; this suggests a reduction in salt intake.

## 1. Introduction

Hypertension is a cardiovascular disease-related risk factor that increases the risk of death [1]. It takes into consideration approximately 10% (1.8 million) of annual cardiovascular disease-related deaths worldwide [2]. Cardiovascular disease can be significantly reduced by moderately reducing dietary salt intake [3]. Thus, salt reduction is of great importance. Japan is one of the countries with a high salt intake and has set target daily salt intakes of <7.5 g for men and <6.5 g for women [4]. According to a 2019 national survey, the salt intake of both men and women in Japan was approximately 3 g higher than the target (men: 10.9 g, women: 9.3 g) [5], which is approximately twice the amount recommended by the World Health Organization (<5 g/day) [6]. Thus, salt reduction is a public health priority for the Japanese population. Approximately 60% of Japanese people who consume ≥8 g of salt have no interest in or intention to improve their diet [5]. Thus, an approach to reduce salt intake without difficulty is needed, even if individuals have little interest in improving their health or diet.

In recent years, many interventions have used nudges, defined as “a behaviorally informed intervention usually made by changing the choices presented to an individual (i.e., choice architecture), which alters people’s behavior in a predictable way” [7], to improve dietary behavior [8,9,10,11]. Studies have classified nudges that promote healthy eating behaviors into three levels: cognitively oriented nudge, such as “descriptive nutritional labeling”, “evaluative nutritional labeling” or “visibility enhancements”; (2) affectively oriented nudge, such as “hedonic enhancements“ or “healthy eating calls”; or (3) behaviorally oriented nudge, such as “convenience enhancements” or “size enhancements” [10]; among the three levels, behaviorally oriented nudges have the greatest effect [10].

Nudges with ingenious utensils are related to behaviorally oriented nudges. In a study comparing the amount of sugar added to tea with a regular spoon and a small spoon, 27% less sugar consumption was reported when the small spoon was used [12]. In addition, the amount of food consumed was lower when eating with a small spoon than when eating with a regular spoon [13]. In a study that compared the amount of four different foods taken from a salad bar with a spoon and tongs, the results showed that the amount taken was 16.5% lower when tongs were used than when spoons were used [14]. The mechanism is that these foods can be scooped more efficiently with spoons. The findings of this study suggested that energy intake can be controlled by serving low-calorie foods with spoons and high-calorie foods with tongs [14]. This study suggested that ease of eating affects the amount of food eaten. A study conducted in Thailand [15] on fish sauce consumption reported that using a specially designed spoon with a hole induced a larger reduction (0.58 g) in the fish sauce used per bowl compared to the normal condition where fish sauce is served in a bottle.

Therefore, we centered our research on the effect of salt reduction by changing eating utensils to reduce salt consumption. We focused on ramen, which is commonly eaten by Japanese people [16]. Ramen is a noodle dish originating from China and contains wheat noodles, sliced pork, seaweed, bean sprouts, and other ingredients in its soup. Its salt content is high [17]; both the noodles and the soup contain salt [18]. In Japan, ramen is usually eaten with chopsticks and spoons without perforations. Chopsticks mainly grasp noodles and ingredients, while spoons scoop the soup and ingredients. Therefore, we hypothesized that if the regular spoon were changed to a perforated spoon, the salt intake would be reduced because ingredients submerged in the soup could be caught efficiently without scooping out the soup. Additionally, it would be a difficult task to scoop the soup with a perforated spoon when trying to eat it, thereby reducing the consumption of the soup. The aim of this randomized crossover study was to evaluate the effect of using a perforated spoon on salt intake by comparing salt intake from a ramen test meal using chopsticks and a regular spoon versus that using chopsticks and a perforated spoon.

## 2. Materials and Methods

### 2.1. Experimental Method

#### 2.1.1. Participants

**Figure 1:**
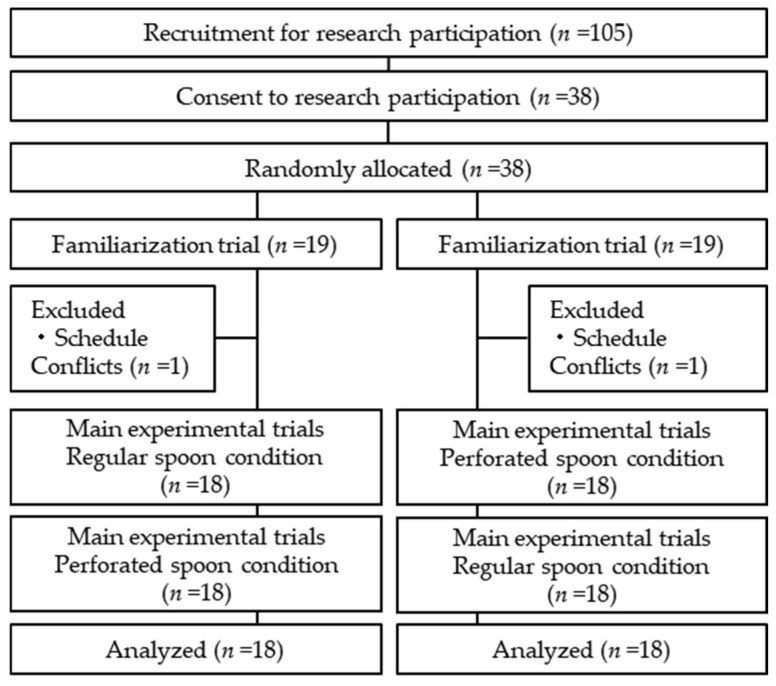
Flow diagram illustrating the flow of participant selection in each of the randomized crossover trial.

The sample size (*n* = 34) was determined based on the expected difference in salt reduction (1.0 ± 2.0 g) using the perforated spoon determined from a pretest conducted beforehand (alpha error = 0.05, power = 0.8) and calculated using G*Power 3.1.9.2 software (Heinrich Heine Universität Düsseldorf, Düsseldorf, Germany). The effect size (Cohen’s d) was calculated to be 0.5. However, we initially recruited 38 participants in anticipation of multiple dropouts. To recruit participants for the study, the authors explained the study to university students on campus; 38 of the 105 students agreed to participate. A flow diagram of participant selection has been illustrated in Figure 1.

The inclusion criteria were as follows: (1) healthy male university students with no acute or chronic morbidities; (2) those not on a diet for weight loss and salt reduction; and (3) non-smokers. Male university students were considered for the study because many young people in Japan are indifferent about maintaining a healthy lifestyle [5], and salt intake is higher among men than among women [5].

This study was conducted in accordance with the principles of the 1964 Declaration of Helsinki and its later amendments. Ethical considerations included explaining to the participants that their participation in the study was voluntary, that they could withdraw their consent at any time, and that written informed consent would be obtained from all participants in advance. The study protocol was approved by the ethics committee of the Prefectural University of Kumamoto (approval number: 04-19).

#### 2.1.2. Study Procedures

In this randomized crossover study, the familiarization trial and main experimental trials were conducted from January to March 2023. There were three trials: a familiarization trial and two main experimental trials. Each experimental trial, including the familiarization trial, was conducted at least 7 days apart. All trials were conducted at lunchtime (12.00–13.00 h), with the time standardized for each participant.

In the familiarization trial, participants ate a lunch test meal of ramen noodles with chopsticks and a regular spoon to familiarize themselves with the laboratory environment. In the subsequent two main experimental trials, the participants ate for lunch ramen with chopsticks and a regular spoon (hereinafter referred to as the regular spoon condition) and with chopsticks and a perforated spoon (hereinafter referred to as the perforated spoon condition) in random order. Participants were randomly allocated to two groups using a random number generator to adjust for order effects. To blind participants to the true aim of the study, we conducted the experiment without informing them of the purpose of the study because informing them of the purpose and content before the start of the experiment may change their eating behavior. The authors who conducted the experiments and measured the results were not blinded.

Participants, including those in the familiarization trial, were asked to refrain from moderate or strenuous exercise and to not consume alcohol from the day before the study until the time of the study, and to spend time as usual. On the day of the main experimental trials, the participants were instructed to replicate the breakfast and morning activity patterns on the day of the familiarization trial. Some participants habitually ate breakfast every day, while others did not. Therefore, participants were instructed that their morning routine should be the same as that on the familiarization trial day, including eating breakfast. Those who ate breakfast on the day of the familiarization trial were instructed to eat breakfast before the two main tests. Participants were instructed to eat breakfast as usual, without any instructions on what to eat. Those not accustomed to eating breakfast were asked to participate in the study without eating breakfast.

The familiarization and main experimental trials were conducted in a room with a separate eating area where the participants could not see what others were eating. Before being offered the test meal and drinking water, participants responded to questions about their appetite (hunger and fullness) and how they spent the morning (e.g., breakfast intake, snacking, and physical activity). Participants were given standardized instructions to “eat ramen as usual.” No instructions were given on how to use the spoon. After the meal, participants were asked about their subjective appetites. They were instructed to remain in the study room until they responded to the questions regarding their subjective appetite after meals.

#### 2.1.3. Test Meal

The test meal was a ramen noodle dish that contained wheat noodles, sliced pork, seaweed, bean sprouts, and other ingredients in the soup. It was selected because it is frequently consumed in Japan [15]. According to a study examining the food-based dietary patterns of Japanese people for breakfast, lunch, and dinner, “ramen” is frequently consumed for during lunch [16]. This study showed that 1504 lunches from the 4-day dietary records of 392 Japanese adults aged 20–69, of which 99 were observed to contain ramen [15]. Generally, ramen has a high salt content [17]. The known salt content per dish is 5.5 g for ramen noodles [19].

The ingredients and nutritional values of the test meal are shown in Table 1. Based on a typical restaurant meal, the energy and salt equivalent of ramen noodles were 459 kcal of energy and 6.9 g of salt equivalent (0.7 g for noodles and ingredients and 6.2 g for soup). The ramen soup was soy sauce flavored and was adjusted using a commercial product (4901468-232063, Kikusui CO, Hokkaido, Japan). The salt equivalent of the noodles was calculated by multiplying the weight of the noodles with the salt content ratio (0.2%) when the noodles were boiled. Drinking water was served in 200 mL portions at the same time as the test meal. Water refills were available throughout the meal, with glasses weighed before the meal as well as after the meal to calculate the amount of water consumed.

#### 2.1.4. Spoons

**Figure 2:**
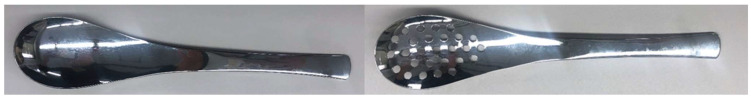
Regular spoon and perforated spoon.

The regular spoon and perforated spoon used in the study are shown in Figure 2. The length of the tip of the regular spoon (Shimomura Kihan CO, Niigata, Japan) and perforated spoon (Shimomura Kihan CO, Niigata, Japan) was 180 mm and 177 mm, respectively, with a width of 37 mm for both. The perforated spoon had 27 holes. The amount of soup that could be scooped in one spoonful by a regular spoon was approximately 9.0 g. The perforated spoon could scoop out the ingredients but not the soup because the soup falls through the holes.

### 2.2. Survey Items

#### 2.2.1. Physical Measurements

During the familiarization test, the participants’ height was measured to the nearest 0.1 cm and the participants’ weight was measured to the nearest 0.1 kg, using a digital scale (TBF-102; Tanita Corporation, Tokyo, Japan). Body mass index was calculated as kg/m^2^.

#### 2.2.2. Subjective Evaluation

Visual analog scales (VAS) ranging from 0 cm to 10 cm were used to evaluate the participant’s appetite (hunger and fullness) before and after meals. For example, in the case of hunger, the right end was defined as “very hungry” and the left end as “not hungry at all,” each evaluated with a 10-cm line. The VAS was evaluated in increments of 0.1 cm. The same method was used to evaluate the taste of the ramen by rating its deliciousness after eating. In the case of deliciousness, the right end was defined as “very delicious” and the left end as “not delicious at all.” Ratings closer to 10.0 indicated more hunger, fullness, and deliciousness (better taste).

#### 2.2.3. Salt Intake, Quantity of the Meal Consumed, Water Consumption and Meal Time

The amount of salt intake and quantity of the meal consumed was calculated by subtracting the amount of noodles, ingredients, and soup remaining at the end of the meal (g) from the amount of noodles, ingredients, and soup provided (g). Water intake was calculated by subtracting the remaining weight from the amount provided. Participants were instructed to present the “meal finished” tag at the end of the meal. The meal time was defined as the time from when the meal was served to the participant (start of the meal) to when the “meal finished” tag was presented (end of the meal).

### 2.3. Statistical Analyses

The Wilcoxon rank-sum test was used to compare the subjective evaluations, salt intake, and quantity of the meal consumed between both spoon conditions. Nonparametric tests were used for salt and soup intake because normality could not be confirmed via histograms and Shapiro–Wilk test (*p* < 0.05). The statistical software SPSS Statistics (version 25.0 for Windows, IBM. Corp, Armonk, NY, USA) was used, and the significance level was set at 5% (two-tailed).

## 3. Results

### 3.1. Participant Characteristics

Of the 38 male participants who agreed to participate in the study, 2 withdrew for scheduling reasons, and 36 were included in the analysis. The average age of the participants was 20.7 (SD 1.8) years, and the mean height and weight were 172.6 (SD 6.2) cm and 65.5 (SD 10.2) kg, respectively. The mean body mass index was 22.0 (SD 2.8) kg/m^2^.

### 3.2. Comparison of Subjective Evaluation before and after Eating under Both Spoon Conditions

Table 2 shows the results of the comparison of subjective evaluations before and after meals. There were no significant differences in hunger or fullness before and after meals under either spoon condition (*p* > 0.05). There was also no significant difference in the evaluation of the deliciousness of the test meals (*p* > 0.05).

### 3.3. Comparison of Salt Intake, Quantity of Meal and Water Consumed, and Meal Time under Both Spoon Conditions

The results of the comparison of salt intake, quantity of meal consumed, quantity of water consumed, and meal time under both spoon conditions are presented in Table 3. The median (25th and 75th percentile) mass of ramen soup intake was significantly lower (*p* = 0.020) in the perforated spoon condition (65.5 [45.9, 204.3] g) than in the regular spoon condition (97.5 [63.7, 227.2] g). Total salt intake (soup, ingredients, and noodles) was also significantly lower (*p* = 0.019) in the perforated spoon condition (1.8 [1.5, 4.3] g) than that in the regular spoon condition (2.4 [1.8, 4.8] g). The amount of water consumed during the meal and meal time did not differ between both spoon conditions (*p* > 0.05). Five participants (14% of those analyzed) consumed all the soup in both conditions.

## 4. Discussion

In this study, we examined the effect of using a perforated spoon on salt intake using ramen as a test meal in men who are young and healthy. The results of the study showed that using a perforated spoon for noodles reduced the amount of salt intake by decreasing the amount of soup consumed compared to using a regular spoon. There were no differences in post-meal fullness or deliciousness between both spoon conditions. Salt intake was lower when the ramen was consumed with the perforated spoon than when consumed using a regular spoon.

This result was attributed to the fact that eating ramen with a perforated spoon reduced the amount of soup broth consumed. The median difference in soup intake between both spoon conditions was approximately 30 g, indicating that the participants consumed approximately three spoons less soup with the perforated spoon. This result supports the hypothesis that using perforated spoons can reduce salt intake by decreasing soup intake. In a previous study [15], an average of 1.38 g of fish sauce was added per serving of noodles, and the amount of fish sauce added was reduced to 0.80 g by providing a specially-designed spoon, reporting a 51.0-mg (approximately 0.13 g of salt; sodium [g] × 2.54) reduction in sodium per serving of noodles. The use of perforated spoons in this study demonstrated a valuable result, with a median difference of 0.6 g in salt intake between both spoon conditions.

The effect of salt reduction using a perforated spoon was confirmed for all participants. However, some (*n* = 5 [14%]) consumed all the soup under both conditions, and no effect of using the perforated spoon was observed. These participants drank it directly from the bowl without using the spoon. Therefore, there was no effect of using the spoon on salt reduction. Those who ate the soup may need to change their spoons and use other strategies to reduce salt intake.

A new finding from this study was that using a perforated spoon in a study of young, healthy male participants with ramen as a test meal reduced salt intake by reducing soup intake without sacrificing the feeling of fullness and taste after the meal. It has been suggested that ramen may increase the risk of stroke mortality due to its high salt content [20]. Making the perforated spoon instead of regular spoons the default option in ramen restaurants and cafeterias may help reduce salt intake from noodle meals, enabling even persons uninterested in reducing their salt intake to do so naturally. Strategies to reduce salt intake by devising such eating utensils, together with the popularization of salt reduction knowledge [21,22], will enhance the effectiveness of salt reduction.

This study had some limitations which should be described. First, it was conducted in an experimental setting, which may have differences from normal eating behavior. Second, the participants were limited to male university students. Therefore, the results cannot be generalized to women or other age groups. Third, the participants were university students, which is a relatively well-educated population. Higher education has been reported to be negatively correlated with salt intake, and the participants in this study may have consumed less soup than the general population [23]. Fourth, we were not able to assess the participants’ usual dietary habits or salt intake. The characteristics of the participants could have been identified to further discuss the external generalization of the results of our study. Fifth, salt intake was calculated based on the weight of the leftover noodles, ingredients, and soup. The amount of soup absorbed by the noodles was not considered. However, since there was no difference in the amount of noodles and ingredients eaten under both spoon conditions, the results were not considered to be significantly affected. Despite these limitations, the results of our study suggest that using a perforated spoon reduces the amount of soup consumed by young men, thereby reducing salt intake.

## 5. Conclusions

This study aimed to test the hypothesis that eating ramen noodles with a perforated spoon would result in less salt intake by reducing the amount of soup consumed compared to eating ramen noodles with a regular spoon without perforation. These results suggest that using a perforated spoon may reduce the salt intake of men who are young and healthy by decreasing the amount of soup consumed by this population. Changes in spoon design may be a new public health measure for reducing salt intake, including for persons indifferent to their health. Future research is required to examine whether using a perforated spoon will have a similar effect on salt reduction for women and other age groups.

## Figures and Tables

**Table 1:** Test meal: ramen ingredients and nutritional value.

	Weigh (g)	Energy (kcal)	Protein (g)	Fat (g)	Carbohydrate (g)	Salt Equivalent (g)
Wheat noodles (boiled) ^(1)^	209	311	10.2	2.5	61.0	0.4
Ramen soup	345	98	3.1	6.0	6.2	6.2
Sliced pork	15	40	2.2	2.9	0.7	0.3
Bean sprouts	50	6	0.9	0.1	1.3	0
Green onion	5	3	0.1	0	0.3	0
Seaweed	0.5	1	0.2	0	0.2	0
Total	624.5	459	16.7	11.5	69.7	6.9

^(1)^ The salt equivalent of Chinese noodles was the amount of salt after boiling the noodles.

**Table 2:** Comparison of subjective evaluations before and after eating under both spoon conditions.

	Before Meals (*n* = 36)	*p* ^(1)^	After Meals (*n* = 36)	*p* ^(1)^
Hunger (cm)				
Regular spoon condition	7.2 (6.1, 8.4)	0.846	2.0 (1.0, 2.5)	0.922
Perforated spoon condition	7.4 (6.5, 8.0)		2.0 (1.0, 3.0)	
Fullness (cm)				
Regular spoon condition	2.4 (1.4, 3.0)	0.277	8.0 (7.1, 9.0)	0.568
Perforated spoon condition	2.3 (1.4, 3.8)		8.0 (7.2, 8.8)	
Deliciousness (cm)				
Regular spoon condition			9.0 (7.5, 10.0)	0.958
Perforated spoon condition			9.0 (8.0, 9.5)	

Values: median (25th and 75th percentile values). Appetite (hunger and fullness) and deliciousness were assessed by subjective psychological ratings using a 0.0–10.0-cm visual analog scale. The visual analog scale was evaluated in increments of 0.1 cm. Ratings closer to 10.0 indicated more hunger, fullness, and deliciousness (better taste). ^(1)^ Wilcoxon rank-sum test.

**Table 3:** Comparison of salt intake, quantity of the meal consumed, quantity of water consumed, and meal time under both spoon conditions.

	Regular Spoon Condition (*n* = 36)	Perforated Spoon Condition (*n* = 36)	*p* ^(1)^
Salt intake			
Whole meal (g)	2.4 (1.8, 4.8)	1.8 (1.5, 4.3)	0.019
Ramen soup (g)	1.7 (1.1, 4.0)	1.1 (0.8, 3.6)	0.018
Noodles and ingredients (g)	0.7 (0.7, 0.7)	0.7 (0.7, 0.7)	0.460
Quantity of the meal consumed			
Whole meal (g)	359.0 (338.3, 496.3)	354.0 (318.8, 473.5)	0.105
Ramen soup (g)	97.5 (63.7, 227.2)	65.5 (45.9, 204.3)	0.020
Noodles and ingredients (g)	267.8 (262.9, 279.9)	272.0 (262.3, 283.5)	0.470
Quantity of water consumed (g)	199 (84, 200)	199 (100, 200)	0.231
Meal time (seconds)	286 (260, 385)	310 (257, 347)	0.652

Values: median (25th and 75th percentile values). ^(1)^ Wilcoxon rank-sum test.

## Data Availability

The data presented in this study are available on request from the corresponding author.
